# A Cascaded RPA-SDA Amplification Strategy on a Sliding Microfluidic Chip for the Ultrasensitive and Rapid Detection of *Shigella*

**DOI:** 10.3390/foods14223891

**Published:** 2025-11-14

**Authors:** Nali Zhou, Guorong Fan, Nan Yang, Tao Xu, Yunlong Zhang, Wentao Xu, Ying Shang

**Affiliations:** 1 Faculty of Food Science and Engineering, Kunming University of Science and Technology, Kunming 650500, China; 2 Key Laboratory of Precision Nutrition and Food Quality, Department of Nutrition and Health, China Agricultural University, Beijing 100193, China

**Keywords:** cascaded amplification, sliding microfluidic chip, DNAzyme, visual detection, *Shigella*

## Abstract

In this study, a sliding microfluidic biosensor integrating RPA-SDA cascaded amplification was developed for the rapid, visual detection of *Shigella*. A novel RPA primer targeting the specific *ipaH* gene was designed to include a 5′-end G-quadruplex (G4) sequence and the complementary sequence of an Nt.BstNBI endonuclease recognition site. The RPA product templates a subsequent SDA reaction, generating abundant G4 structures that form peroxidase-mimicking DNAzymes with hemin, catalyzing a TMB reaction that produces a distinct blue color for visual readout (on-chip detection at OD_370_, distinct from conventional tube assays at OD_450_). The core on-chip detection process was completed within 13 min (10 min for SDA and 3 min for color development), achieving a limit of detection of 3.5 × 10^−4^ ng/μL for *Shigella* genomic DNA. This timing explicitly excludes the preceding, off-chip steps of nucleic acid extraction and RPA amplification. Validation using spiked lettuce samples confirmed the platform’s high specificity and sensitivity. This work establishes a proof-of-concept for a portable screening tool, highlighting its potential for on-site food safety applications. However, further validation in diverse food matrices and under real-world field conditions is required to fully establish its practical utility.

## 1. Introduction

*Shigella*, a Gram-negative bacterium transmitted via contaminated food or water, is the causative agent of bacillary dysentery. The severe inflammatory disease damages the colon and ileum, posing a significant public health risk, particularly in developing countries where it impairs childhood development [1,2,3]. Effective control is compounded by the spread of antimicrobial resistance, creating an urgent demand for rapid and sensitive diagnostic tools [4,5]. However, traditional methods are inadequate for point-of-care diagnostics [6]. Bacterial culture is prohibitively slow and cannot detect viable but non-culturable (VBNC) cells [7]. Immunoassays suffer from cross-reactivity with other enteric pathogens [8], and PCR-based techniques require bulky, expensive equipment and complex handling, precluding their use in on-site settings [9]. Isothermal amplification techniques have emerged as powerful alternatives to conventional PCR, offering rapid and equipment-free nucleic acid analysis suitable for Point-of-Care Testing (POCT), which help address several of the limitations mentioned above [10]. Notably, recombinase polymerase amplification (RPA) and strand displacement amplification (SDA) have emerged as powerful alternatives due to their exceptional speed and efficiency, making them ideal for field-deployable applications [11,12,13].

RPA technology introduced by Piepenburg et al. [14] operates at a low, constant temperature, using specific primers and probes to ensure high sensitivity and specificity [9,15], which is particularly suitable for providing an important technical support for POCT [16,17]. Likewise, Strand Displacement Amplification (SDA) functions isothermally (for example 37–42 °C), eliminating the need for thermal cyclers and enabling diverse detection methods for its products, such as lateral flow or fluorescence [18,19].

Despite their individual strengths, single isothermal methods can still suffer from limitations, including inadequate sensitivity or the need for complex readout instrumentation. To overcome these challenges, cascaded amplification systems have been developed. These advanced strategies synergistically combine the strengths of multiple techniques to dramatically improve overall detection sensitivity and specificity, enhance reaction efficiency, and minimize the risk of non-specific amplification, representing a significant step forward for molecular diagnostics [20,21,22]. Despite their power, a critical gap prevents the widespread use of cascaded amplification for on-site *Shigella* detection: the reliance on non-integrated, pump-driven systems and functionally limited primers that prevent a direct link between amplification and a visual signal. This study directly addresses this limitation by constructing a sliding-based, pump-free microfluidic chip. This device establishes an integrated ‘amplification-signal output’ system—from reagent storage to final detection—all made possible by a specially engineered primer that drives both the amplification and the visual readout on-chip.

Biosensor technology is pivotal for enabling the on-site detection of foodborne pathogens, offering key advantages in good portability, ease of operation, and rapid detection. These devices are broadly classified by their signal output mechanism into electrochemical, optical, piezoelectric, and thermal types [23,24,25,26]. Among these, colorimetric biosensors are especially well-suited for field applications, as their visual output allows for straightforward result interpretation without reliance on complex instrumentation [27]. A prevalent strategy in colorimetric sensing involves the use of G-quadruplex (G4)/hemin DNAzymes. These complexes function as peroxidase mimics, catalyzing the oxidation of a chromogenic substrate (for example ELISA chromogenic substrate) to produce a distinct color change. This mechanism provides both inherent signal amplification and a direct visual readout, making it a powerful tool for sensitive detection [28,29,30]. To realize the full potential of this approach, these sensing principles are often integrated into microfluidic “lab-on-a-chip” platforms. Microfluidics allows for the precise handling of samples and reagents in miniaturized channels, seamlessly coupling the biomolecular recognition event with signal generation. This integration facilitates the development of rapid, automated, and highly sensitive sample-to-answer systems for point-of-care diagnostics [31,32,33].

This study introduces a sliding microfluidic biosensor designed for the visual, on-site detection of foodborne pathogens, which overcomes key bottlenecks in both biochemistry and device integration. A common challenge in cascaded amplification is the ‘separation of functional modules,’ which complicates workflows. Our core biochemical innovation addresses this directly: we engineered a single RPA primer with a unique all-in-one design, featuring a 5′-end containing both a G4 sequence and an Nt.BstNBI recognition site. This specialized sequence engineering allows the RPA product to directly template an SDA reaction, establishing a seamless ‘amplification-to-detection’ system that generates a visual signal without needing separate probes. To implement this strategy in a user-friendly format, we designed an integrated, two-layer sliding microfluidic chip. The upper layer houses pre-loaded reagents in separate chambers, effectively minimizing manual pipetting steps. A simple sliding motion aligns these chambers with the lower layer, creating a single, continuous fluidic path. This mechanism not only initiates the reaction but also ensures homogenous mixing, while simultaneously reducing reagent volume, preventing cross-contamination, and enhancing overall assay stability.

Through systematic optimization, the biosensor achieved a low limit of detection (LOD) and a broad dynamic range for the demonstrated target. Beyond this specific application, the platform’s architecture holds strong potential for adaptability. However, repurposing it for other targets would require more than a simple primer swap; it would involve a careful re-design of the target-specific primers, including optimization of the Nt.BstNBI cut-site context to ensure efficient cleavage, and potentially fine-tuning reaction conditions. This inherent versatility, combined with its all-in-one design ideal for POCT, positions the device as a promising and adaptable framework for developing future on-site diagnostics.

## 2. Materials and Methods

### 2.1. Materials and Reagents

Bacterial strains: *Shigella* (CMCC(B) 51572) and (ATCC 12022), *Escherichia coli* O157:H7 (NCTC 12900), *Bacillus cereus* (ATCC 14579), *Listeria monocytogenes* (CMCC(B) 54002), *Staphylococcus aureus* (CMCC(B) 26003), and *Salmonella enterica* (CMCC(B) 50115) were all purchased from Luwei Technology Co., Ltd. (Shanghai, China).

Kits and enzymes: The TwistAmp^®^ Basic RPA reaction kit was obtained from TwistDx (Cambridge, UK). The Ezup Columnar Bacterial Genomic DNA Extraction Kit and the ELISA chromogenic substrate (TMB) were purchased from Sangon Biotech (Shanghai) Co., Ltd. (Shanghai, China). Bst DNA Polymerase 2.0 and Nt.BstNBI endonuclease were purchased from Gene Biotechnology International Trade Co., Ltd. (Shanghai, China).

Chemicals and other materials: Hemin was purchased from Macklin Biochemical Co., Ltd. (Shanghai, China). Sulfuric acid (H_2_SO_4_) was purchased from Kelong Chemical Co., Ltd. (Chengdu, China). The lettuce was purchased from the local Zhuanxin Farmers’ Market (Yunnan, China).

Sliding microfluidic chip: The sliding microfluidic chip was fabricated from Polydimethylsiloxane (PDMS) by the School of Environmental and Biological Engineering, Putian University. The device featured circular chambers with a diameter of 8 mm and a depth of 160 μm, resulting in a nominal reagent volume of 8 μL per chamber. These chambers were interconnected by channels with a uniform width of 600 μm. No specific surface treatments were applied to the PDMS. For assembly, a thin layer of mineral oil was applied between the two PDMS layers to create a reversible, leak-proof seal, and the device was then secured with external clips. The chips were manufactured to a precise specification, with intra- and inter-batch volumetric variations controlled to less than 5%, ensuring the reproducibility required for the assay.

### 2.2. Instruments and Equipment

Portable Centrifuge: Model DE1008E (Shanghai Keya Biotechnology Co., Ltd., Shanghai, China); Thermostatic Mixing Block: Model JXH-100 (Tuohe Electromechanical Technology Co., Ltd., Shanghai, China); Multi-functional Microplate Reader: Model LUX-3020 (Kunming Youning Technology Co., Ltd., Kunming, China); Benchtop High-Speed Centrifuge: (Eppendorf AG, Hamburg, Germany).

### 2.3. Experimental Procedures

#### 2.3.1. Genomic DNA Extraction and Quality Verification

Genomic DNA (gDNA) was extracted from 1 mL of an overnight *Shigella* culture, grown in LB broth and harvested at an optical density (OD600) of approximately 1.5 (~1.5 × 10^9^ CFU/mL), using the Ezup Columnar Bacterial Genomic DNA Extraction Kit following the manufacturer’s protocol. The concentration and purity of the extracted gDNA were quantified using a NanoDrop 2000 spectrophotometer(Thermo Fisher Scientific, Wilmington, DE, USA), with purity assessed via the A260/280 and A260/230 absorbance ratios. DNA integrity was evaluated on 1% agarose gel to confirm the presence of high-molecular-weight DNA without significant degradation. Furthermore, successful extraction of amplifiable DNA was validated by PCR targeting the 16S rDNA gene with universal primers [34], using the following conditions: 94 °C for 5 min; 30 cycles of denaturation at 94 °C for 30 s, 58 °C for 30 s, and 72 °C for 30 s; and a final extension at 72 °C for 10 min. The amplicons was visualized on a 2% agarose gel, with the presence of a single, sharp band of the expected size serving as the criterion for use in subsequent assays. DNA samples that met these quality standards were stored at −20 °C.

#### 2.3.2. Selection and Validation of the Target Gene and Primer Design

Candidate target genes for the specific detection of *Shigella* were first identified through a comprehensive literature review (Table S1). The specificity of these candidate genes was then experimentally validated through a multi-step process. Initially, specificity was screened against *E. coli* O157:H7 as a negative control. Genes that showed no cross-reactivity were further tested against a panel of non-target bacteria, including *S. aureus*, *Salmonella*, *L. monocytogenes*, and *B. cereus*. A target gene that consistently amplified only in *Shigella* (CMCC(B) 51572) was selected for further evaluation. The inclusivity of this selected gene was then confirmed by demonstrating consistent amplification in a different strain, *Shigella* (ATCC 12022). A gene was selected as the definitive target for this study based on its performance across our screening panel. In repeated assays, it demonstrated both no observable cross-reactivity against the selected non-target bacteria and consistent positive amplification for all tested *Shigella* strains. Specific RPA primers were then designed based on the sequence of this validated gene for subsequent assays. Based on the sequence of this validated gene, specific RPA primers were designed for the subsequent assays.

Based on the sequence of the selected target gene, *ipaH* (GenBank Accession: OQ955245.1), candidate primers were designed and screened in silico for potential secondary structures using the IDT PrimerQuest™ tool (https://www.idtdna.com/pages/tools/primerquest (accessed 26 December 2023)) to select sequences with minimal risk of self-dimers and hairpins (Table S2). All oligonucleotides were commercially synthesized with PAGE purification (Sangon Biotech, Co., Ltd., Shanghai, China). The in silico specificity of each primer set was rigorously verified via BLASTn analysis against the NCBI nucleotide database to ensure minimal off-target hybridization. Subsequently, the performance of these candidate primers was experimentally evaluated using both conventional PCR and RPA assays, with the products analyzed on 2% agarose gel. The optimal primer set was selected based on its ability to consistently produce a single, clear band of the expected size, with no non-specific amplification or primer-dimer formation. This dual-system screening confirmed the primers’ robustness and suitability for the subsequent development of the RPA-SDA cascade amplification system.

#### 2.3.3. Design of Primers for the RPA-SDA Cascade System

Based on the preceding validation, the *ipaH-c* primer set was selected as the foundation for the final assay primers. To construct the RPA-SDA cascade detection system, the core sequences of the *ipaH-c* primers were extended at their 5′ ends with two functional motifs: the complementary sequence of the Nt.BstNBI endonuclease recognition site (5′-GACTC-3′) and the complementary sequence of the PW17 G-quadruplex (5′-AACCCAACCCGCCCTACCC-3′) [35]. This engineering process, which also incorporated a poly-thymidine spacer (TTTTT) for flexibility, resulted in the final, fully modified primers used throughout this study. The complete sequences are as follows, *ipaH-SDA-F*: 5′-AACCCAACCCGCCCTACCCTTTTGACTCGGCAGCCTGGTTTCCTGAAGCAGATCGTCG-3′; *ipaH-SDA-R*: 5′-AACCCAACCCGCCCTACCCTTTTTGACTCCGGAGGTATTGCGTGCAGAGACGGTATCGG-3′. This integrated primer design ensures that the initial RPA amplicons carry the necessary recognition sites and templates for the subsequent isothermal SDA reaction and signal generation.

#### 2.3.4. Optimization of the RPA-SDA Cascade Reaction

To improve the performance of the *Shigella* RPA-SDA assay, we conducted a systematic optimization of its core parameters. The process began with an initial set of baseline reaction conditions, which are detailed in Tables S3–S5. Subsequently, a one-variable-at-a-time approach was employed to refine this baseline protocol. Each optimization experiment was performed in triplicate (n = 3). For each parameter, the condition that yielded the highest mean absorbance value with the lowest standard deviation was selected as optimal. This selection was based on observing a clear, non-overlapping separation in the mean ± standard deviation values when compared to sub-optimal conditions. Key parameters for both the RPA and SDA stages were varied across the following ranges:

1. RPA parameter optimization: primer (10 μmol/L): 0, 0.6, 1.2, 2.4, 3.6, 4.8, 6.0, 7.2, 8.4, 9.6, and 10.8 μL; reaction temperature: 27, 30, 33, 36, 39, 42, 45, and 48 °C; reaction time: 5, 10, 15, 20, 25, 30, and 35 min; MgOAc (280 mmol/L): 0, 0.5, 1.0, 1.5, 2.0, 2.5, 3.0, 3.5, 4.0, and 4.5 μL.
2. SDA parameter optimization: enzyme ratio (Bst 2.0/Nt.BstNBI): 2:1, 3:2, 1:1, 5:6, 2:3, and 1:2; reaction temperature: 56, 58, 60, 62, 64, 66, and 68 °C; reaction time: 5, 10, 15, 20, 25, and 30 min; dNTP mixture (10 mmol/L each): 0, 2, 4, 6, and 8 μL; MgSO_4_ (100 mmol/L): 0, 0.5, 1.0, 1.5, 2.0, 2.5, 3.0, 3.5, and 4.0 μL.

The outcome of each optimization experiment was evaluated using a colorimetric assay. The principle of this assay is that the G4 structures within the SDA amplification products can, in the presence of hemin, catalyze the oxidation of TMB by H_2_O_2_, producing a colored solution. To do this, after SDA, the reaction was continued to be incubated at 95 °C for 20 min to inactivate the enzyme and terminate the reaction, and the 25 µL SDA product was mixed with 2 µL of 2 µM Hemin. Subsequently, 36.5 µL of TMB color development solution and 36.5 µL of H_2_O_2_ solution were added to the mixture. After a 3 min incubation at room temperature to allow for color development, the reaction was terminated by adding 23.1 µL of 2 M H_2_SO_4_. Quantitative analysis was achieved by immediately measuring the absorbance at 450 nm (OD_450_) with a microplate reader. The condition that resulted in the highest absorbance reading was identified as the optimal setting for that parameter.

#### 2.3.5. Specificity and Sensitivity of the RPA-SDA Cascade Reaction

To validate the specificity of the RPA-SDA cascade reaction, a panel of non-target bacteria, including *Escherichia coli* O157:H7, *Salmonella*, *Bacillus cereus*, *Listeria monocytogenes*, and *Staphylococcus aureus*, was tested alongside the target organism, *Shigella*. Genomic DNA was extracted from overnight cultures of each strain to serve as the template for amplification. The reactions were performed under the previously optimized RPA-SDA conditions. Assay performance was evaluated through a dual-assessment method: qualitatively, by recording the visual color change of the TMB reaction, and quantitatively, by measuring the absorbance at 450 nm with a microplate reader. This approach provided a comprehensive evaluation of the cascade system’s ability to specifically recognize *Shigella*.

The analytical sensitivity of the RPA-SDA cascade reaction was subsequently determined using a 10-fold serial dilution of purified *Shigella* genomic DNA, starting from an initial concentration of 30 ng/μL. This generated a series of templates with final concentrations of 30, 3, 0.3, 0.03, 0.003, and 0.0003 ng/μL. Each of these dilutions was amplified using the optimized protocol. Similar to the specificity testing, the results were analyzed both visually and by absorbance measurement at 450 nm to determine the limit of detection for the RPA-SDA detection system. The LOD was determined empirically and is defined as the lowest concentration of target DNA that consistently yielded a positive signal clearly distinguishable from the negative control across three independent replicates.

#### 2.3.6. Design and Optimization of the Sliding Microfluidic Chip System

A custom-designed sliding microfluidic chip (50 mm × 40 mm) was used for all on-chip assays. The device features an innovative two-layer structure: a slidable upper module with four independent storage chambers and a fixed lower module with four corresponding reaction chambers. This design integrates the amplification and detection steps into a single, enclosed platform.

The standard operational protocol was as follows: First, a thin layer of silicone oil was applied to the lower chip surface to enhance sealing and ensure smooth movement. The upper and lower modules were then precisely aligned. For each reaction, reagents were loaded sequentially into a storage chamber: 20 μL of the SDA premix, 2 μL of hemin solution, and 3 μL of the RPA amplification product. Concurrently, 5 μL of the TMB/H_2_O_2_ substrate was added to the corresponding reaction chamber. The entire chip was then sealed with a transparent film to prevent evaporation and contamination.

To adapt the assay for this microfluidic format, which notably eliminates the need for a sulfuric acid stop solution, the on-chip reaction conditions were systematically optimized. The process involved a 10 min SDA incubation, followed by sliding the upper module to mix the amplicon with the TMB substrate to initiate the colorimetric reaction. To determine the optimal endpoint for this final step, two key parameters were evaluated. First, the color development time was assessed at intervals of 30, 60, 90, 120, and 180 s. Subsequently, using the optimal time identified, the reaction temperature was optimized by testing incubation at 54, 58, 62, and 66 °C. For on-chip analysis, the microfluidic device was placed directly into a standard microplate reader. Measurements were taken using a vertical optical path, with the light beam passing through the center of the reaction chamber. Absorbance was recorded at 370 nm. To circumvent the corrosive effects of sulphuric acid on the chip materials and streamline the detection process, this system employs a strategy of direct reading without terminating the reaction. Consequently, the detection wavelength was adjusted from 450 nm (for the acid-stopped product) to the characteristic absorption peak of the non-terminated blue TMB reaction product at 370 nm. For both optimization experiments, the ideal condition was selected based on the strongest signal determined by visual assessment and quantitative absorbance measurement at 370 nm.

#### 2.3.7. Validation of Specificity and Sensitivity of the Sliding Microfluidic Chip

The performance of the sliding microfluidic chip system was validated by assessing its specificity and sensitivity, following the general methodology established in Section 2.3.5. For the specificity evaluation, the same panel of target (*Shigella*) and non-target bacterial DNA was tested using the optimized on-chip protocol. The outcome was assessed by direct visual observation and absorbance measurement at 370 nm to check for potential cross-reactivity.

To determine analytical sensitivity, a 10-fold serial dilution of *Shigella* DNA, starting from a concentration of 35 ng/μL, was prepared and analyzed on the chip. The limit of detection (LOD) was to be determined by identifying the lowest template concentration that yielded a consistent positive signal.

#### 2.3.8. Validation of the Chip System with Spiked Lettuce Samples

As a proof-of-concept, the detection capability of the sliding microfluidic chip system was evaluated in a complex food matrix, using lettuce as a representative sample. Commercially available lettuce was first washed with deionized water, homogenized into a slurry, and then sterilized by autoclaving (121 °C for 20 min) to eliminate background microbial contamination. The sterilized lettuce was then divided into five experimental groups and artificially contaminated (spiked). To validate the assay’s feasibility in a complex food matrix, sterile lettuce homogenate was spiked with pathogen genomic DNA to a final concentration of 3.5 ng/μL, the groups were as follows: 1, a negative Control (un-spiked); 2, a *Shigella* positive group (spiked with *Shigella* culture); 3, a non-target control (spiked only with *S. aureus*); 4, a co-infection group (spiked with a 1:1 mixture of *Shigella* and *Staphylococcus aureus* cultures); 5, an interference group (spiked with *Shigella* in the presence of *E. coli* O157:H7 culture).

After spiking, all samples were incubated at 37 °C for 12 h to simulate contamination. To recover the bacteria, 5 g of each sample was vigorously mixed with 45 mL of PBS buffer to release the bacterial cells. The suspension was then centrifuged at 5000 rpm for 10 min to pellet the cells. Genomic DNA was subsequently extracted from the pellets using an Ezup column bacterial genomic DNA extraction kit according to the manufacturer’s protocol. This extracted DNA served as the template for the on-chip analysis under the previously optimized conditions. The final colorimetric reaction was developed using the TMB substrate at 37 °C for 15 min. The outcome was assessed both visually and by measuring the absorbance at 370 nm with a microplate reader.

## 3. Results

### 3.1. Principle and Functional Characterization of the Integrated Microfluidic System

**Figure 1:**
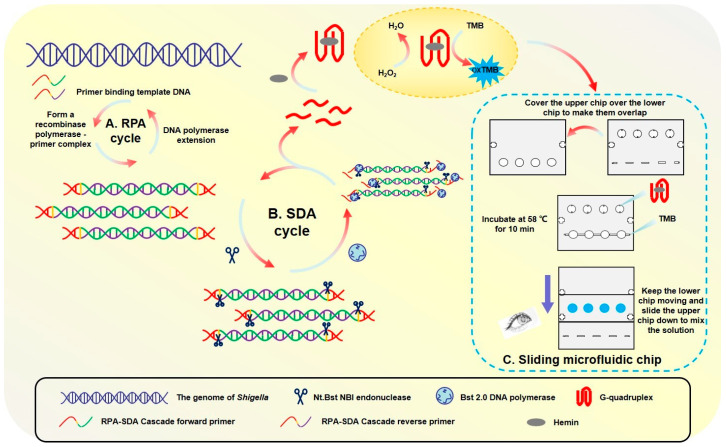
Specific process of sliding microfluidic biosensing.

The primary outcome of this work was the development and characterization of a rapid, integrated biosensing system. The principle and physical embodiment of this system are illustrated in Figure 1.

The detection is based on an RPA-SDA enzymatic cascade. In essence, the process uses an initial RPA step to generate templates that fuel a subsequent SDA reaction, producing a large quantity of single-stranded G-quadruplex DNA. These DNA strands act as the key signaling output; they fold and bind with hemin to form a peroxidase-mimicking DNAzyme, which catalytically converts a TMB substrate into a distinct blue product.

To execute this principle in a user-friendly format, a novel sliding microfluidic chip (50 mm × 40 mm) was constructed (Figure 1C). The chip’s innovative two-layer design physically integrates the SDA reaction and the colorimetric detection into a single, self-contained platform. The upper slidable module holds the initial reagents, while the fixed lower module contains the reaction chambers. As shown in Figure 1A, this design enables a simple, two-step workflow: amplification, the chip is incubated for 10 min at 58 °C to generate the G-quadruplex molecules; detection, a manual sliding action merges the amplification product with the TMB substrate, initiating a 3 min color development reaction at the same temperature.

The novelty of this work lies in the strategic integration of a cascaded RPA-SDA isothermal amplification, a functionally engineered primer design, and a user-friendly microfluidic chip, which collectively enable the rapid, ultrasensitive, and visual detection of *Shigella*.

The high specificity of the proposed assay is ensured by a multi-level mechanism. Firstly, the RPA primers exclusively target the *Shigella*-specific *ipaH* gene. Secondly, only the correct amplicons contain the necessary functional motifs (G4 sequence and endonuclease site) to initiate the SDA reaction and subsequent signal generation, effectively preventing false-positive signals from non-target DNA.

This integrated design proved to be highly efficient, successfully translating the complex biochemical process into a simple procedure. The functional characterization confirmed that the entire detection workflow, from loading the sample to obtaining a clear, visual color change, can be completed in under 15 min, demonstrating its potential for rapid, point-of-care diagnostics.

### 3.2. Optimization of the RPA-SDA Cascade Reaction

To maximize the analytical performance of the RPA-SDA cascade assay, key parameters for both the initial amplification stage (RPA) and the signal amplification stage (SDA) were systematically optimized.

For the initial RPA stage, optimization focused on achieving high amplification efficiency while minimizing non-specific byproducts, with outcomes analyzed by agarose gel electrophoresis. A primer volume of 2.4 μL yielded the brightest target band, ensuring sufficient template binding. In contrast, higher concentrations led to the formation of obvious primer-dimers (Figure S1). The optimal reaction time and temperature were determined to be 25 min (Figure S2) and 39 °C (Figure S3), respectively; shorter times resulted in incomplete amplification, while longer durations or higher temperatures led to decreased band intensity, likely due to enzyme activity decline or substrate depletion. The optimal amount of magnesium ion addition was found to be 1.5 μL (Figure S4), as concentrations beyond this point caused the amplification efficiency to first plateau and then significantly decrease.

**Figure 2:**
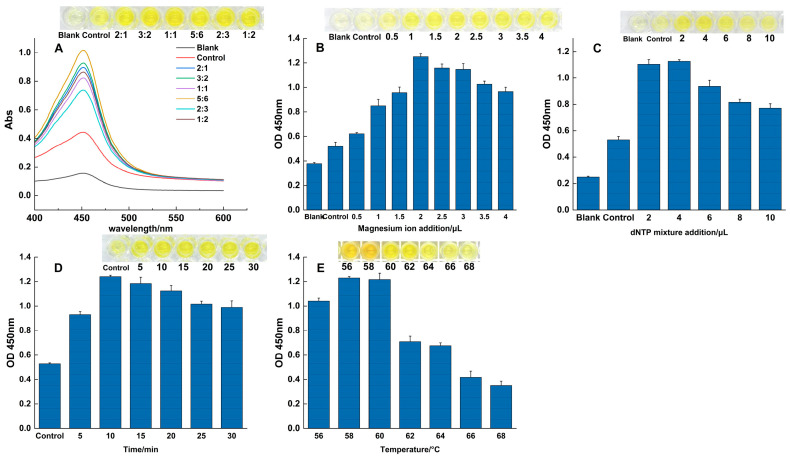
Optimization of the SDA reaction. The effects of varying key parameters on the colorimetric signal (OD_450_), including: (**A**) the activity ratio of the two enzymes, (**B**) magnesium ion concentration, (**C**) dNTP concentration, (**D**) reaction time, and (**E**) reaction temperature.

Following RPA, the parameters for the SDA signal amplification step were optimized using colorimetric analysis, with results quantified by absorbance at 450 nm. The results are presented in Figure 2. Enzyme Ratio: The activity ratio of Bst 2.0 DNA polymerase to Nt.BstNBI endonuclease was critical for balancing the reaction kinetics. An enzyme activity ratio of 5:6 produced the strongest signal, indicating optimal equilibrium between the nicking and extension steps (Figure 2A); key reagents: further optimization identified the ideal conditions as 2 μL of MgSO_4_ (Figure 2B) and 4 μL of the dNTP mixture (Figure 2C), as concentrations outside these values led to significant inhibition or reduced efficiency; time and temperature: a reaction duration of 10 min was determined to be optimal, at which point the accumulation of the product reached a plateau (Figure 2D). The temperature gradient experiment confirmed that 58 °C was the ideal reaction temperature, as signal intensity decreased significantly beyond this point (Figure 2E). All optimization experiments were performed in triplicate. The established parameters from this comprehensive optimization process formed a robust and reliable protocol for all subsequent experiments.

### 3.3. Specificity and Sensitivity of the RPA-SDA Cascade Reaction

The analytical performance of the developed RPA-SDA assay was rigorously evaluated in terms of its specificity and sensitivity.

**Figure 3:**
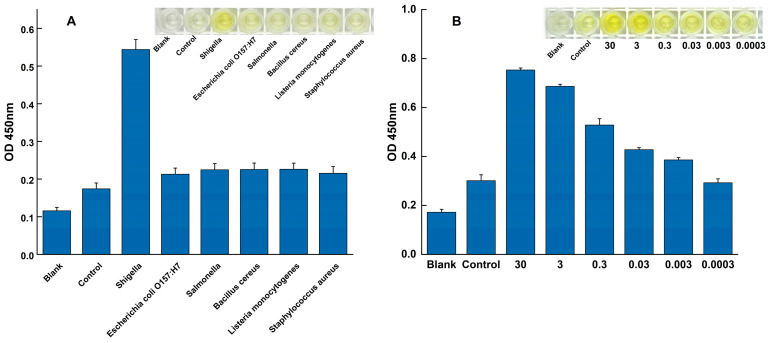
Specificity and sensitivity of the RPA-SDA cascade amplification assay. (**A**) Specificity was validated against *Shigella* (positive control) and a panel of non-target pathogens. (**B**) Sensitivity was evaluated using a serial dilution of *Shigella* genomic DNA to determine the limit of detection.

The specificity of the assay was evaluated against a panel of common foodborne pathogens to assess its ability to exclusively detect *Shigella*. As shown in Figure 3A, all specificity tests were performed in triplicate (n = 3). A strong, distinct positive signal was observed exclusively when *Shigella* genomic DNA was used as the template. In contrast, the absorbance signals from all tested non-target pathogens were indistinguishable from the negative control, with their mean values and error bars (mean ± SD) overlapping with the baseline. This result confirms the assay’s high specificity for *Shigella*, effectively distinguishing it from other related microorganisms.

The analytical sensitivity was determined by applying the assay to a serial dilution of *Shigella* genomic DNA under the fully optimized reaction conditions. A clear dose-dependent response was observed (Figure 3B). The LOD was established as 3 × 10^−3^ ng/μL, as this was the lowest concentration where a distinct positive signal was consistently detected across all replicates (n = 3). At this LOD concentration, the resulting mean absorbance value and its standard deviation (mean ± SD) were clearly separated from the negative control baseline, providing the necessary precision. Conversely, at the next dilution level (3 × 10^−4^ ng/μL), the signal was indistinguishable from the negative control.

### 3.4. Optimization and Validation of the Sliding Microfluidic Chip System

To adapt the assay for the miniaturized format and ensure maximal signal intensity and reliability, the colorimetric detection conditions on the sliding microfluidic chip were systematically optimized.

**Figure 4:**
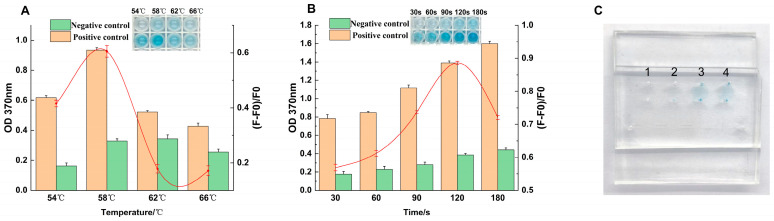
Optimization and visual results of the sliding microfluidic chip assay. Optimization of reaction temperature (**A**) and reaction time (**B**) for the on-chip assay. (**C**) Visual demonstration of the chip’s performance showing the results for a blank (lane 1), a negative control (lane 2), and two parallel positive samples (lane 3 and 4) under the final optimized conditions.

The results of the color development time optimization are presented in Figure 4A. As the reaction time increased, the absorbance value at 370 nm (OD_370_) showed a corresponding increase. The optimal signal intensity was achieved at 120 s. At this time point, the background-deducted signal reached its maximum, indicating the best balance between a strong positive signal and minimal non-specific interference, resulting in a distinct blue color.

Using this optimal reaction time, the effect of temperature on the color development efficiency was then evaluated (Figure 4B). The system’s performance peaked when the reaction temperature was 58 °C, as evidenced by the highest OD_370_ value and background-deducted signal. It was noted that when the temperature exceeded 58 °C, the signal intensity began to decrease, likely due to the adverse effects of a high-temperature environment on the stability or activity of the DNAzyme. Based on these results, the finalized optimal conditions for all subsequent on-chip experiments were established as a 120 s color development step performed at 58 °C.

Our proposed RPA-SDA assay offers significant operational advantages over standard methods like qPCR. By operating at a single constant temperature, it reduces the total assay time to under 40 min and eliminates the need for a specialized thermal cycler. To validate its practical utility for POCT, we then implemented this optimized assay on a microfluidic chip and compared it to the conventional 96-well plate format (Figure 4C). The on-chip system not only introduced further efficiencies, such as reduced reagent consumption, but also maintained a fully comparable detection performance. Crucially, it preserved the key analytical metrics established for the method: the high sensitivity (LOD of 3 × 10^−3^ ng/μL, Figure 3B) and the high specificity with no false positives against non-target pathogens (Figure 3A). This successful adaptation confirms the robustness of the optimized conditions on the miniaturized platform and validates its value for rapid and reliable screening.

### 3.5. Specificity and Sensitivity Validation of the Sliding Microfluidic Chip System

To confirm the practical performance of the integrated device, the specificity and sensitivity of the sliding microfluidic chip system were rigorously validated.

**Figure 5:**
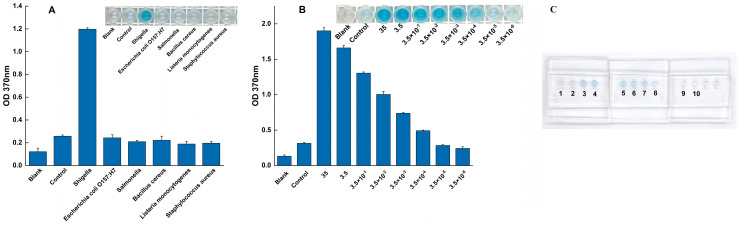
Specificity and sensitivity performance of the sliding microfluidic chip. (**A**) Specificity of the assay was confirmed against a panel of non-target foodborne pathogens, with *Shigella* serving as the positive control. The assay’s sensitivity was evaluated both quantitatively and visually; (**B**) A calibration curve showing the colorimetric response (OD_370_) to serial dilutions of *Shigella* genomic DNA. (**C**) The corresponding visual results on the microfluidic chip, where lanes 3–10 contain decreasing concentrations of target DNA (from 35 down to 3.5 × 10^−5^ ng/μL), alongside a blank (1) and a negative control (2).

The specificity of the on-chip assay was tested against a panel of common non-target pathogens. As demonstrated in Figure 5A, the results were definitive. The non-target strains produced no discernible colorimetric reaction, with their absorbance values remaining at a low baseline level, identical to the negative control. In sharp contrast, the chip produced a significant colorimetric signal and a high absorbance value exclusively for *Shigella*. This confirms that the high specificity of the assay is fully maintained within the microfluidic system, with no evidence of cross-reactivity.

The on-chip LOD was determined using a serial dilution of *Shigella* genomic DNA. The results, shown in Figure 5B, indicate that the system could reliably detect the target down to a concentration of 3.5 × 10^−4^ ng/μL, producing a clear color reaction and a signal unambiguously distinct from the negative control. At the next dilution (3.5 × 10^−5^ ng/μL), the signal was indistinguishable from the background, thus establishing the LOD at 3.5 × 10^−4^ ng/μL.

This on-chip sensitivity not only matches but slightly improves upon the performance of the conventional 96-well plate assay. The successful and reproducible validation of both specificity and sensitivity strongly supports the fundamental reliability and practical value of this integrated microfluidic system. While further studies are needed to quantify inter-chip variation and long-term reagent stability, these results demonstrate a robust foundation for its use in rapid and accurate pathogen detection.

### 3.6. Validation of the Assay in Spiked Food Samples

**Figure 6:**
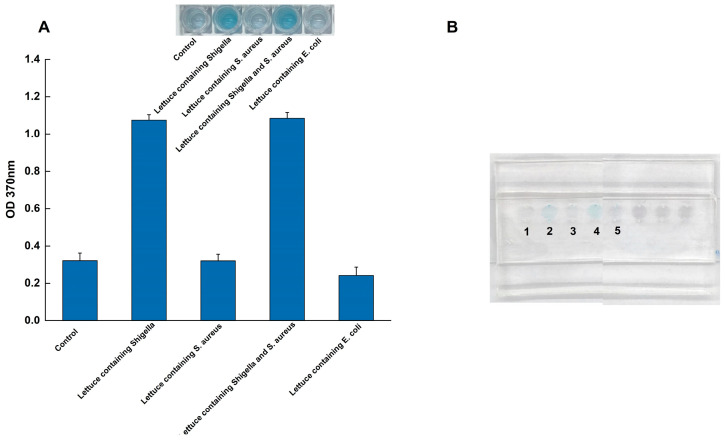
Validation of the microfluidic system using spiked lettuce samples. (**A**) Quantitative absorbance readings (OD_370_); (**B**) The corresponding visual results on the chip, tests were performed on a negative control (lane 1) and lettuce samples spiked with *Shigella* (lane 2), *S. aureus* (lane 3), a mixture of *Shigella* and *S. aureus* (lane 4), and *E. coli* (lane 5) to demonstrate practical applicability and specificity in a complex food matrix.

To evaluate the practical applicability of the sliding microfluidic chip system, its performance was tested using artificially contaminated lettuce samples (Figure 6). Lettuce homogenate was spiked with *Shigella* at a concentration of 3.5 ng/μL (genomic DNA). To challenge the assay’s specificity in an interference test, a high concentration of a competing microorganism, *S. aureus*, was simultaneously added at 3.5 ng/μL (genomic DNA).

The system demonstrated excellent performance under these conditions. As shown in Figure 6, control samples containing only the lettuce matrix yielded no reaction, confirming that food components did not trigger false positives. In contrast, the system achieved a 100% hit rate (n = 3 replicates), successfully detecting *Shigella* in all spiked samples. Crucially, this detection was unaffected by the high concentration of *S. aureus*, with no observable interference. Taken together with the specificity results, the successful detection amidst a high concentration of competing bacteria and complex food matrix components underscores the system’s strong anti-interference capabilities.

While the ability to perform accurately despite potential food-based inhibitors and competing DNA highlights its significant potential for on-site screening, we acknowledge this proof-of-concept study was limited to a single food matrix. Further validation in other matrices, such as meat or dairy, is a key objective for future work.

## 4. Discussion

In this study, we successfully developed and validated an integrated sliding microfluidic chip system for the rapid, highly sensitive, and specific detection of *Shigella*. This platform provides an efficient solution for on-site pathogen screening, addressing a critical need in food safety. The discussion below will focus on the technical innovations, performance advantages, and practical application potential of this system.

1. Technical Innovation and Design Advantages

A primary innovation of this work is the synergistic cascade of two isothermal amplification techniques—RPA and SDA—integrated with a visually clear colorimetric readout. RPA’s rapid, low-temperature (39 °C) operation serves as an ideal primary amplification stage, while the subsequent SDA reaction (58 °C) provides a powerful secondary signal amplification. This signal is generated through the release of G-quadruplex-forming DNA sequences, which assemble with hemin to create a DNAzyme that catalytically oxidizes TMB, producing a distinct blue color. This eliminates the need for complex analytical instrumentation. It is worth noting that differences in wavelength and timing between tubular reactions and chip-based assays, alongside potential stability fluctuations within the G-quadruplex/heme DNAase chemical system, necessitate systematic optimisation and evaluation to ensure the feasibility of subsequent translational applications.

The dual-layer sliding microfluidic chip is another key design advantage. This simple, pump-free mechanism allows for sequential reagent mixing via a single manual action, dramatically simplifying the workflow. From a design standpoint, the enclosed and miniaturized reaction chambers are intended to minimize the risk of aerosol-based cross-contamination. This is a critical consideration, as carryover between samples is a known factor that can compromise the stability and reproducibility of nucleic acid amplification assays. The strategic engineering of the primers to contain both the Nt.BstNBI recognition site and the G-quadruplex sequence was fundamental to ensuring the efficiency and specificity of this cascade.

2. Superior Detection Performance

The optimized system demonstrated excellent analytical performance. The on-chip assay achieved a LOD of 3.5 × 10^−4^ ng/μL for *Shigella* genomic DNA. This sensitivity is nearly an order of magnitude higher than that of the same assay performed in a conventional 96-well plate format (LOD of 3.0 × 10^−3^ ng/μL). Furthermore, specificity tests confirmed that the system accurately distinguished *Shigella* from a panel of other common foodborne pathogens with no observable cross-reactivity.

While PCR is the gold standard, its reliance on thermal cyclers limits its field utility. In contrast, our system is inherently designed for on-site use by integrating rapid, instrument-free isothermal amplification with simple visual detection on a portable microfluidic chip.

At the field application level, this system demonstrates significant advantages: the entire detection process can be completed within 13 min without relying on bulky electrically powered equipment such as thermal cyclers, greatly enhancing operational convenience in the field. Through validation using spiked lettuce samples, this method successfully achieved accurate detection of the target pathogen, confirming its practical application potential in on-site food safety monitoring. However, to transition this proof-of-concept into a fully validated, field-deployable tool, several aspects must be addressed in future work. First, the use of autoclave-treated lettuce—while deliberately chosen for this initial study to establish a clear baseline—is a limitation. Second, nucleic acid extraction is currently an external process, and the method has only been validated for a single matrix. Therefore, subsequent research will focus on two primary goals: developing an integrated sample-to-answer system, and validating its performance across a variety of fresh, non-sterilized food samples. This comprehensive validation will be essential for assessing the method’s real-world robustness and for performing a full quantitative analysis, including the systematic determination of its recovery efficiency and limit of quantification (LOQ).

Finally, it is worth noting that several technical aspects warrant systematic optimisation to ensure the feasibility of subsequent translational applications. Future work should therefore include: (1) expanding specificity testing against a more comprehensive non-target panel; and (2) addressing minor inconsistencies in signal timing and stability, which may stem from differences between reaction formats and the inherent variability of the G-quadruplex/hemin catalytic chemistry.

3. Future Outlook and Potential for Industrialization

While this system offers clear advantages in speed, simplicity, and cost, we acknowledge areas for further development. The primary opportunity lies in creating a true sample-to-answer device by integrating an on-chip sample preparation module. Future work should also focus on expanding the platform’s capability for multiplex detection to simultaneously screen for several pathogens. Finally, incorporating intelligent integration, such as using a smartphone application to read and quantify the colorimetric signal, would enhance data management and user-friendliness.

In conclusion, through a combination of innovative molecular strategy and user-centric microfluidic design, this study has produced a powerful diagnostic platform. Its demonstrated high sensitivity, specificity, and robustness confirm its significant potential for commercialization and widespread application, particularly for on-site food safety screening.

## 5. Conclusions

In this study, we successfully developed and validated an integrated sliding microfluidic chip that combines a novel RPA-SDA cascade amplification with a simple colorimetric readout for the detection of *Shigella*. This system provides a powerful and practical solution for rapid, point-of-care food safety testing. The key achievement of this work is the creation of a highly efficient assay that achieves a limit of detection of 3.5 × 10^−4^ ng/μL in under 15 min. This high performance is realized through the synergistic combination of two isothermal amplification methods and a G-quadruplex/hemin DNAzyme-catalyzed visual signal, which completely eliminates the need for complex instrumentation and significantly lowers the barrier to use. The innovative, pump-free sliding chip design proved to be robust and user-friendly, successfully detecting *Shigella* even in complex food matrices. This confirms its reliability for real-world applications. By establishing a platform that is simultaneously rapid, sensitive, specific, and easy to operate, this research provides a valuable tool for decentralized food safety monitoring. The principles demonstrated here can be readily adapted to target other pathogens, holding significant promise for preventing foodborne disease outbreaks and advancing molecular diagnostics in resource-limited settings.

## Data Availability

The original contributions presented in the study are included in the article/Supplementary Material, further inquiries can be directed to the corresponding author.

## Supplementary Materials

The following supporting information can be downloaded at: https://www.mdpi.com/article/10.3390/foods14223891/s1, Figure S1: Optimization results of primer addition in RPA reaction system. Figure S2: The result of time optimization of RPA reaction system. Figure S3: The result of temperature optimization of RPA reaction system. Figure S4: Optimization results of magnesium ion addition in RPA reaction system. Table S1: Primer sequence of *Shigella* target gene. Table S2: Sequence of RPA candidate primers for *Shigella*. Table S3: RPA reaction steps. Table S4: SDA reaction systems. Table S5: TMB color reaction system.

## Abbreviations

The following abbreviations are used in this manuscript:

RPA	recombinase polymerase amplification
TMB	ELISA chromogenic substrate
G4	G-quadruplex
SDA	Strand Displacement Amplification
POCT	Point-of-Care Testing
VBNC	viable but non-culturable
LOD	limit of detection
