# Supplementary material for: A Cascaded RPA-SDA Amplification Strategy on a Sliding Microfluidic Chip for the Ultrasensitive and Rapid Detection of Shigella
Source: Foods. 2025 Nov 14;14(22):3891. doi: 10.3390/foods14223891 (PMC12651771; doi:10.3390/foods14223891)
Supplement: Supplementary file 1 [file foods-14-03891-s001.zip › foods-3959760-supplementary.pdf]

Supplementary Material

Table S1 Primer sequence of *Shigella* target gene

Primer name	Primer sequence（5'-3'）	Product length (bp)
acrR -F	AAG TCG GAT TTG TTC AGT G	564
acrR -R	ATT CGT TAG TGG CAG GAT	
marOR -F	CAC TCT TTA GCT AGC CTT G	604
marOR -R	TGG ACA TCG TCA TAC CTC T	
gyrA -F	TAC ACC GGT CAA CAT TGA GG	648
gyrA -R	TTA ATG ATT GCC GCC GTC GG	
parC -F	GTC TGA ACT GGG CCT GAA TGC	248
parC -R	AGC AGC TCG GAA TAT TTC GAC AA	
ial-F	GGA GTG GTA TGG ATG GTC	320
ial-R	CCA GGC CAA CAA TTA TTT CC	
sheT1-F	GTG AAC CTG CTG CCG ATA TC	147
sheT1-R	ATT TGT GGA TAA AAA TGA CG	
sheT2-F	CAT AAT AAT AAG CGG TCA GC	798
sheT2-R	ATG TGC CTG CTA TTA TTT AT	
ipaH1-F	GCT GGA AAA ACT CAG TGC CT	200
ipaH1-R	CCA GTC CGT AAA TTC ATT CT	
ipaH2-F	CAT GGC TGG AAA AAC TCA GTG C	512
ipaH2-R	CTC ATA CTT CTG CTC TTC TGC C	
ipaH3-R	TCC GGA GAT TGT TCC ATG TG	420
ipaH3-R	TGT ATC ACA GAT ATG GCA TGC	
ipaH4-R	GGA TTC CGT GAA CAG GTC G	595
ipaH4-R	CGC TCA GAC CTG ATG CTT TCA	

Table S2 Sequence of RPA candidate primers for *Shigella*

Primer name	Primer sequence (5'-3')	Product length (bp)
ipaH-aF	CCT TTT CGA TAA TGA TAC CGG CGC TCT GCT	213
ipaH-aR	GTC ACT CCC GAC ACG CCA TAG AAA CGC ATT	
ipaH-bF	CCT TGG CGC TTT CCT CGC TAC CTG TAC TCC	237
ipaH-bR	CTG CGC TTA GTG ATT TGA TGG TGT CTG GTA	
ipaH-cF	GGC AGC CTG GTT TCC TGA AGC AGA TCG TCG	137
ipaH-cR	CGG AGG TAT TGC GTG CAG AGA CGG TAT CGG	

Table S3 RPA reaction steps

Number	Operation steps	Add reagents and perform the operation	Addition amount (μL)/condition
1	Prepare the premix in a 1.5 ml centrifuge tube	Primer Free Rehydration Buffer	29.5
		Forward primer (10 μM)	2.4
		Reverse primer (10 μM)	2.4
		Template DNA	2.5
		ddH_2O	Supplement the reaction system to 47.5
2	Vortex oscillation	Briefly vortex the prepared premix	
3	Mix freeze-dried enzyme powder	Transfer the premix to the freeze-dried enzyme powder tube and vortex it again	
4	Add MgOAc	Drop MgOAc (280 mM) into the tube cap	2.5
5	Instantaneous centrifugation	Ensure uniform mixing after instantaneous centrifugation	
6	Reaction incubation	Place the reaction tube in a constant-temperature mixed metal bath	39 ℃，25 min

Table S4 SDA reaction systems

Reactive component	Addition amount (μL)	Reaction conditions
RPA product	5	Incubate at 62℃ for 15 minutes Then incubate at 95℃ for 20 minutes. Inactivate the enzyme and terminate the reaction.
dNTP mixture	4	
$MgSO_4$	2	
1×isothermal ampliflications buffer	2.5	
Bst2.0 DNA polymerase	1.25	
Nt.BstNBI nicking endonuclease	1.5	
ddH_2O	Supplement the reaction system to 25	
In total	25	

Table S5 TMB color reaction system

Reactive component	Addition amount (μL)	Reaction conditions
SDA product	25	After shaking and mixing in a centrifuge, react at room temperature for 3 minutes
Hemin(The final concentration is 2μM)	2	
TMB chromogenic solution	36.5	
H_2O_2 chromogenic solution	36.5	
In total	100	

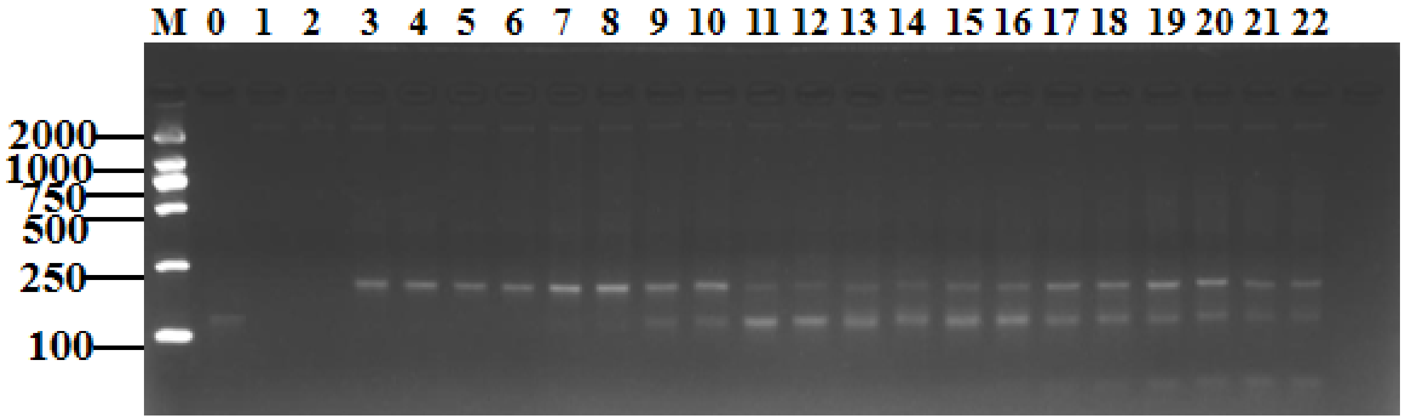

Figure S1 Optimization results of primer addition in RPA reaction system: M: DNA marker DL2000; 0: negative control; 1-2: 0 μL; 3-4: 0.6 μL; 5-6: 1.2 μL; 7-8: 2.4 μL; 9-10: 3.6 μL; 11-12: 4.8 μL; 13-14: 6.0 μL; 15-16: 7.2 μL; 17-18: 8.4 μL; 19-20: 9.6 μL; 21-22: 10.8 μL

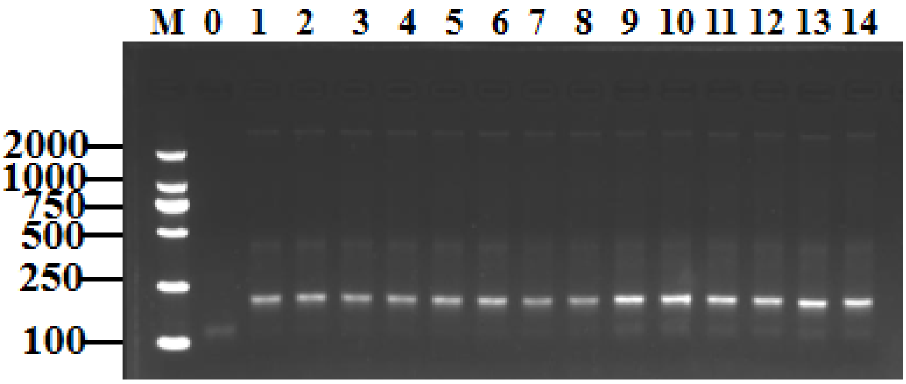

Figure S2 The result of time optimization of RPA reaction system: M: DNA marker DL2000; 0: negative control; 1-2: 5 min; 3-4: 10 min; 5-6: 15 min; 7-8: 20 min; 9-10: 25 min; 11-12: 30 min; 13-14: 35 min

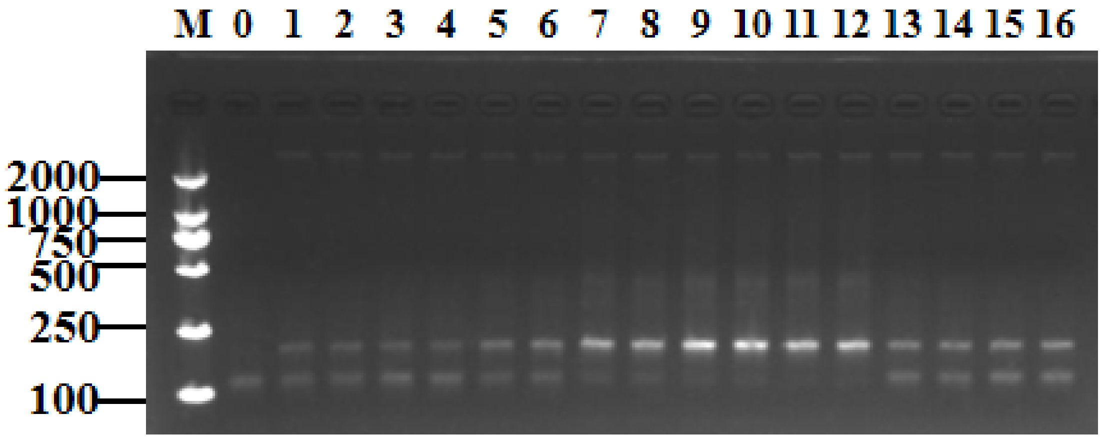

Figure S3 The result of temperature optimization of RPA reaction system: M: DNA marker DL2000; 0: negative control; 1-2: 27℃; 3-4: 30℃; 5-6: 33℃; 7-8: 36℃; 9-10: 39℃; 11-12: 42℃; 13-14: 45℃; 15-16: 48℃

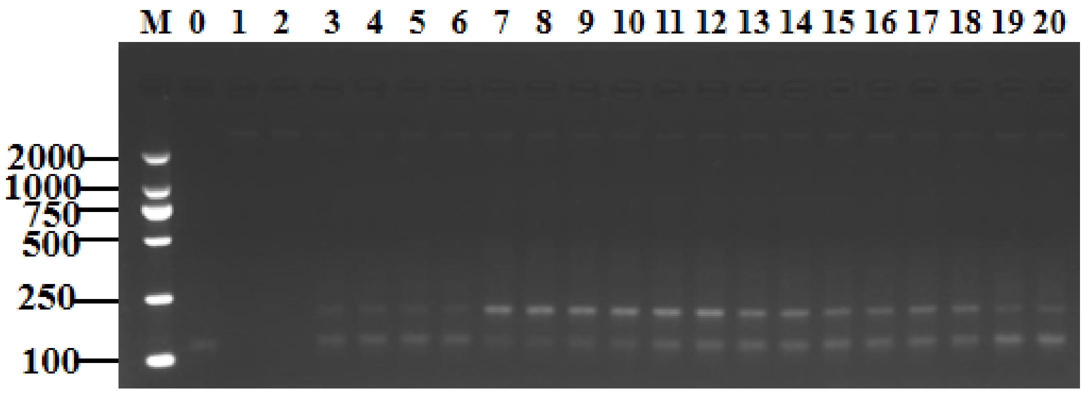

Figure S4 Optimization results of magnesium ion addition in RPA reaction system: M: DNA marker DL2000; 0: negative control; 1-2: 0 μL; 3-4: 0.5 μL; 5-6: 1 μL; 7-8: 1.5 μL; 9-10: 2 μL; 11-12: 2.5 μL; 13-14: 3 μL; 15-16: 3.5 μL; 17-18: 4 μL; 19-20: 4.5 μL
